# Acute Fatty Liver of Pregnancy Presenting With Isolated Nausea and Vomiting in the Third Trimester: A Case Report

**DOI:** 10.7759/cureus.113661

**Published:** 2026-07-30

**Authors:** Sarah Kassar, Cynthia Di Giovanni, Clotilde Lamy

**Affiliations:** 1 Obstetrics and Gynaecology, Erasmus Hospital, Brussels, BEL

**Keywords:** acute fatty liver of pregnancy, case report, obstetric emergency, swansea criteria, third trimester

## Abstract

Acute fatty liver of pregnancy (AFLP) is a rare but life-threatening obstetric emergency occurring in the third trimester. Its non-specific presentation frequently overlaps with other pregnancy-related conditions, leading to diagnostic delay and increased maternal and fetal morbidity and mortality.

We report the case of a 27-year-old primigravida at 38 + six weeks of gestation presenting with isolated nausea and vomiting, without abdominal pain, hypertension, proteinuria, or obstetric complaints. Clinical examination revealed mild jaundice and stable hemodynamics. Initial laboratory investigations demonstrated leukocytosis, hepatic cytolysis, hyperbilirubinemia, coagulation abnormalities, and hypoglycemia. Repeat testing within 10 hours showed rapid biological deterioration with worsening leukocytosis, acute kidney injury, and progressive coagulopathy. In the absence of thrombocytopenia and hypertension, and with fulfillment of eight Swansea criteria, AFLP was strongly suspected. Emergency cesarean section was performed for maternal indication, with prophylactic perioperative coagulation support. A female neonate weighing 2,440 g was admitted briefly to the neonatal intensive care unit for transient respiratory distress. Postpartum maternal recovery was rapid, with normalization of all biological parameters, and both patients were discharged in good condition.

AFLP remains a diagnostic challenge due to its non-specific presentation. Early recognition using the Swansea criteria, prompt delivery, and multidisciplinary supportive care are essential to optimize outcomes. Neonatal metabolic screening for fatty acid oxidation disorders should be systematically considered.

## Introduction

Acute fatty liver of pregnancy (AFLP) is a rare obstetric emergency with an estimated incidence of one in 7,000 to 20,000 pregnancies [1,2]. It represents the leading cause of acute liver failure during pregnancy and typically occurs in the third trimester or early postpartum period. Despite advances in obstetric and intensive care, AFLP remains associated with significant maternal and fetal morbidity and mortality if not promptly diagnosed and treated.

The pathophysiology of AFLP is not fully understood, but is strongly associated with disorders of fetal fatty acid oxidation, particularly deficiencies in long-chain 3-hydroxyacyl-CoA dehydrogenase (LCHAD) [3,4]. Impaired fetal and placental fatty acid metabolism leads to the accumulation of toxic fatty acid intermediates in the maternal circulation, resulting in microvesicular steatosis and hepatocellular dysfunction [3]. Identified risk factors include multiple gestation, low maternal body mass index, male fetus, and a prior history of AFLP, with recurrence reported in up to 20% of subsequent pregnancies [1,2].

Clinical presentation is frequently non-specific and includes nausea, vomiting, malaise, abdominal discomfort, jaundice, and headache [1,2,5]. Notably, abdominal pain is absent in up to 37% of AFLP cases at initial presentation [6]. These symptoms overlap considerably with common pregnancy complaints and other pregnancy-related liver disorders, particularly preeclampsia and Hemolysis, Elevated Liver enzymes, and Low Platelet (HELLP) syndrome, contributing to diagnostic delay [2,7]. Importantly, the absence of classic features such as abdominal pain, hypertension, or thrombocytopenia does not exclude the diagnosis of AFLP and should not delay clinical suspicion or workup.

Liver biopsy demonstrating microvesicular steatosis is diagnostic but is rarely performed due to coagulation abnormalities and the urgency of clinical management [1,2]. Diagnosis therefore relies on clinical and laboratory findings, using the Swansea criteria [8]. The presence of six or more criteria in the absence of an alternative diagnosis strongly supports AFLP [7,8]. Early diagnosis is crucial, as prompt delivery and intensive supportive care have dramatically reduced maternal mortality from over 70% in the 1980s [9], to less than 20% in recent series [2,10,11].

## Case presentation

A 27-year-old pregnant woman, gravida 1, at 38 + six weeks of gestation, presented to the emergency department with nausea and vomiting of recent onset. She reported no abdominal pain, fever, headache, visual disturbances, or obstetric complaints. Her pregnancy was complicated by insulin-dependent gestational diabetes mellitus. Her pre-pregnancy body mass index (BMI) was 25 kg/m². Her medical history included childhood epilepsy without recent seizures. She was taking only prenatal vitamins and insulin.

On admission, vital signs were stable, with a blood pressure of 120/60 mmHg and a heart rate of 95 beats per minute. Physical examination revealed mild jaundice without abdominal tenderness. Obstetric examination was unremarkable.

Initial laboratory investigations revealed leukocytosis (20,160/µL), elevated liver transaminases (AST 148 UI/L, ALT 143 UI/L), hyperbilirubinemia (total bilirubin 7.1 mg/dL), prolonged activated partial thromboplastin time (APTT 40 sec), reduced prothrombin time (PTT 64.4%), elevated C-reactive protein, and reduced fibrinogen (1.46 g/L). Renal function was not assessed at this time point. Complete biological values are presented in Table 1.

**Table 1:** Key laboratory values at multiple timepoints after admission H: hours from admission; WBC: white blood cell count; PTT: prothrombin time; APTT: activated partial thromboplastin time;  AST: aspartate aminotransferase; ALT: alanine aminotransferase; CRP: C-reactive protein.

Parameter	Reference range	H0	H+5	H+10
WBC (×10⁹/L)	4–11	20.16	19.59	22.27
PTT (%)	70–140	64.4	53.7	49.7
APTT (sec)	20–29	40	43.3	45.5
Fibrinogen (g/L)	2–4	1.46	1.18	—
Creatinine (mg/dL)	0.55–1.02	—	1.38	1.5
Uric acid (mg/dL)	3.1–7.8	—	7.1	7.3
Total bilirubin (mg/dL)	0.3–1.2	—	7.1	7
AST/GOT (UI/L)	0–34	148	149	146
ALT/GPT (UI/L)	10–49	143	141	132
Glycemia (mg/dL)	70–100	—	—	67
CRP (mg/L)	0–5	16.1	17.6	18.1

A repeat blood test was performed five hours after admission (H+5), prior to delivery. A further sample collected 10 hours after admission (H+10), in the immediate post-operative period, demonstrated worsening leukocytosis (22,270/µL), progressive coagulation abnormalities (APTT 45.5 sec, PTT 49.7%), and persistent hypoglycemia (67 mg/dL), confirming ongoing biological deterioration despite surgical delivery. Renal function, assessed for the first time at H+5, showed a creatinine of 1.38 mg/dL, rising to 1.50 mg/dL at H+10 (reference range 0.55-1.02 mg/dL). Although the absolute value did not reach the Swansea threshold of 150 µmol/L, the rapid ascending kinetics were consistent with emerging acute kidney injury. Elevated uric acid (7.1 mg/dL at H+5, corresponding to 422 µmol/L, above the Swansea threshold of 340 µmol/L) was also documented [8].

Differential diagnoses included HELLP syndrome and AFLP. The platelet count on admission was 152 × 10⁹/L (within normal range), and the absence of hypertension, proteinuria, and thrombocytopenia made HELLP syndrome less likely [2,7]. Of note, mild jaundice and vomiting - while present in this patient - may also be observed in HELLP syndrome, and do not in themselves allow for differentiation between the two conditions. Hepatic ultrasound was not performed, as it was judged non-contributory to clinical decision-making; studies have shown neither adequate sensitivity nor specificity of ultrasound for the diagnosis of AFLP [7]. The patient fulfilled eight Swansea criteria - vomiting, hyperbilirubinemia, hypoglycemia, hyperuricemia, leukocytosis, elevated transaminases, emerging acute kidney injury, and coagulopathy - strongly supporting a diagnosis of AFLP (Table 2) [7,8].

**Table 2:** Patient assessment as per Swansea criteria ✓: criterion fulfilled; ~: borderline; —: not fulfilled or not assessed. ^*^Hepatic US (ultrasound) was not performed, as it was judged non-contributory to clinical decision-making; studies have shown neither adequate sensitivity nor specificity of ultrasound for the diagnosis of AFLP [7]. ^†^Creatinine of 1.50 mg/dL (133 µmol/L) did not reach the Swansea threshold of 150 µmol/L; however, the ascending kinetics (not dosed at H0, 1.38 mg/dL at H+5, 1.50 mg/dL at H+10) were consistent with emerging acute kidney injury. ^‡^Liver biopsy was not performed due to the urgency of clinical presentation and associated coagulopathy. Six or more criteria in the absence of an alternative diagnosis support AFLP; this patient fulfilled eight criteria.

Swansea criterion	Threshold	Patient value	Fulfilled
Vomiting	—	Present	✓
Abdominal pain	—	Absent	—
Polydipsia / polyuria	—	Absent	—
Encephalopathy	—	Absent	—
Hyperbilirubinemia	>14 µmol/L	Present	✓
Hypoglycemia	<4 mmol/L	Present (67 mg/dL)	✓
Elevated uric acid	>340 µmol/L	Present (7.1 mg/dL = 422 µmol/L)	✓
Leukocytosis	>11×10⁹/L	Present (20,160/µL at H0)	✓
Ascites / bright liver on US	—	Not performed^*^	—
Elevated transaminases	>42 UI/L	Present (AST 148, ALT 143 UI/L)	✓
Hyperammonemia	>47 µmol/L	Not assessed	—
Acute kidney injury	Creatinine >150 µmol/L	Borderline^†^ (133 µmol/L at H+10)	~
Coagulopathy	PT >14s or PTT >34s	Present (APTT 40s at H0; PTT 64.4%)	✓
Microvesicular steatosis on biopsy	—	Not performed^‡^	—

Given the severity and rapid progression of maternal biological abnormalities, an emergency cesarean section was performed seven hours after admission. The decision for surgical delivery was based on maternal indication, primiparity, and an unfavorable cervix. Prophylactic perioperative coagulation support included administration of fibrinogen concentrate, fresh frozen plasma, and platelet transfusion. Although the platelet count was within the normal range on admission (152 × 10⁹/L), prophylactic platelet transfusion was administered given the rapidly evolving coagulopathy and the high hemorrhagic risk associated with emergency cesarean section in the context of AFLP [12]. The procedure was uncomplicated, with an estimated blood loss of 350 mL.

A female neonate weighing 2,440 g was delivered, with Apgar scores of four, six, and seven at one, five, and 10 minutes, respectively. The newborn was admitted to the neonatal intensive care unit for respiratory distress attributed to delayed lung fluid clearance. Glycemic monitoring was performed until day four of life in the context of the mother's gestational diabetes; all values remained within normal limits. Notably, this surveillance was not initiated as part of a structured AFLP-related neonatal screening protocol for long-chain 3-hydroxyacyl-CoA dehydrogenase (LCHAD) deficiency.

Postoperatively, the mother was monitored in the intensive care unit until postoperative day three. Liver function tests improved within 24 hours after delivery. Acute kidney injury resolved spontaneously by postoperative day two, and coagulation parameters normalized by postoperative day seven [7]. The patient was discharged with complete clinical and biological recovery. The clinical course is summarized in Table 3.

**Table 3:** Clinical timeline H: hours from admission; POD: postoperative day; APTT: activated partial thromboplastin time; AFLP: acute fatty liver of pregnancy; NICU: neonatal intensive care unit.

Time	Event
H0 — Admission	Isolated nausea and vomiting. BP 120/60 mmHg, HR 95 bpm. Mild jaundice on examination.
H0 — Initial workup	Leukocytosis, hepatic cytolysis, hyperbilirubinemia, prolonged APTT, hypoglycemia.
H+5	Worsening leukocytosis, progressive coagulopathy, emergence of acute kidney injury (creatinine 1.50 mg/dL).
H+10	Eight of fourteen Swansea criteria fulfilled. AFLP diagnosis established.
H+7	Emergency cesarean section (maternal indication, primiparity, unfavorable cervix).
H+7	Delivery: female neonate, 2,440 g, Apgar 4/6/7. NICU admission for respiratory distress.
POD+1	Improvement of hepatic function tests.
POD+2	Resolution of acute kidney injury.
POD+3	Discharge from intensive care unit.
POD+7	Normalization of coagulation parameters. Maternal discharge.

## Discussion

This case illustrates the diagnostic and management challenges posed by AFLP when it presents with isolated, non-specific gastrointestinal symptoms. The patient had no abdominal pain, no hypertension, no proteinuria, and no thrombocytopenia - all features that are frequently used to suspect more common pregnancy-related conditions such as HELLP syndrome [2,5,13]. Yet within hours, she developed a rapidly evolving picture of multiorgan dysfunction, underscoring how deceptively benign the initial presentation of AFLP can be.

The diagnostic challenge in this case is representative of the broader literature. Beyond the intrinsic overlap with other obstetric conditions, the absence of pathognomonic early symptoms and the limited availability of liver biopsy in the acute setting represent additional diagnostic barriers - particularly in institutions without immediate access to hepatology expertise. AFLP shares clinical features with several other pregnancy-related liver disorders, most notably HELLP syndrome and severe preeclampsia, making differentiation difficult on clinical grounds alone [1,2]. In this patient, the absence of thrombocytopenia, hypertension, and proteinuria helped exclude HELLP syndrome, while the constellation of metabolic derangements - hypoglycemia, hyperuricemia, hyperbilirubinemia, and coagulopathy - was more consistent with AFLP, a pattern also described in other published cases of AFLP presenting without classic features [14]. The Swansea criteria, which do not require histological confirmation, proved to be a practical and efficient diagnostic tool: the patient met eight of the 14 criteria within the first 10 hours of admission (Table 2), enabling timely clinical decision-making [7].

The biological deterioration observed over 10 hours in this case - worsening leukocytosis, progressive coagulopathy, and the emergence of acute kidney injury (Table 1) - reflects the characteristic trajectory of untreated AFLP toward fulminant hepatic failure and multiorgan dysfunction [1,3,15], which may also include acute pancreatitis and intrauterine fetal death in the most severe cases [16]. In the 1980s, maternal mortality from AFLP exceeded 75%, largely because the diagnosis was often made post-mortem [2,9]. Advances in clinical awareness, the adoption of standardized diagnostic criteria, and improvements in intensive supportive care have reduced mortality to an estimated 12-18% in contemporary series [2,11].

The decision to proceed with emergency cesarean section seven hours after admission was driven by the severity of maternal biological deterioration, primiparity, and an unfavorable cervix. Although there is currently no consensus on the optimal mode of delivery in AFLP, cesarean section accounts for approximately 67% of deliveries in reported cases, often for fetal or obstetric indications [12]. Some studies suggest that cesarean delivery may be associated with lower maternal and fetal mortality compared to vaginal delivery; however, patients undergoing surgical delivery may face an increased risk of hemorrhagic complications due to coagulopathy [12]. In this case, prophylactic perioperative administration of fibrinogen concentrate, fresh frozen plasma, and platelet transfusion was essential to minimize this risk, and blood loss remained within acceptable limits.

Maternal recovery following delivery was consistent with the expected postpartum course of AFLP. Liver function tests improved within 24 hours of delivery, acute kidney injury resolved by postoperative day two, and coagulation parameters normalized by day seven. These findings align with published data showing that hepatic tests typically improve within one to two days, coagulopathy resolves within four to six days, and acute kidney injury remits within seven to 10 days after delivery [7]. Complete biological recovery is the norm, even if histological changes may persist for up to five weeks postpartum [7].

From a neonatal perspective, this case highlights an important gap in multidisciplinary management. Approximately 20% of AFLP cases are associated with a homozygous fetal deficiency in LCHAD, most commonly due to the G1528C mutation [3,4]. When the fetus is homozygous for this defect, and the mother is heterozygous, the resulting impairment of maternal fatty acid oxidation contributes to the accumulation of long-chain metabolites in the liver, perpetuating hepatocellular injury [3,4]. LCHAD deficiency in the neonate can manifest primarily as hypoglycemia, cardiomyopathy, and peripheral neuropathy, and early detection is critical to prevent severe neonatal morbidity and mortality [4,11]. In the present case, glycemic monitoring was performed until day four of life and remained within normal limits; however, this surveillance was initiated in the context of the mother's gestational diabetes rather than as part of a structured AFLP-related neonatal screening protocol for LCHAD deficiency. This distinction is clinically important: systematic neonatal metabolic screening - and, if positive, parental genetic testing - should be explicitly triggered in all infants born to mothers with confirmed AFLP, regardless of whether neonatal symptoms are present at birth and regardless of other neonatal indications already in place.

This case report has several limitations. The diagnosis was established on clinical and biological grounds using the Swansea criteria, without histological confirmation by liver biopsy - a standard limitation given the associated coagulopathy and urgency of management. Neonatal metabolic screening for LCHAD deficiency was not systematically performed as part of an AFLP-specific protocol, and no postpartum follow-up data were available. As a single case report, the generalizability of our findings is inherently limited.

## Conclusions

AFLP is a rare but potentially fatal obstetric condition requiring a high index of suspicion. This case illustrates that isolated gastrointestinal complaints in late pregnancy may mask severe hepatic dysfunction with rapid biological deterioration. Early recognition using the Swansea criteria, prompt delivery, and coordinated multidisciplinary supportive care are the cornerstones of management. Neonatal metabolic screening for LCHAD deficiency should be systematically considered in infants born to mothers with confirmed AFLP. Heightened clinical awareness of atypical AFLP presentations is essential to reduce diagnostic delay and prevent severe maternal and fetal complications.
